# A cross sectional study of optical coherence tomography angiography biomarkers and best corrected visual acuity in epiretinal membrane eyes

**DOI:** 10.1186/s40942-026-00855-w

**Published:** 2026-05-09

**Authors:** Shing Chuen Chow, Nicholas Siu Kay Fung, Jeffrey Man Yeung Lo, Kendrick Co Shih, Allie Lee, Angie Hon Chi Fong, Phoebe Cheuk Lam Lam, Lai Yu Ng, Rachel Ya Yuan Leung, Christopher Kai Shun Leung

**Affiliations:** 1 The Li Ka Shing Faculty of Medicine, Department of Ophthalmology, The University of Hong Kong, Room 301, Level 3 Block B, Cyberport 4 100 Cyberport Road, Hong Kong, China; 2 Grantham Hospital, Hong Kong, China

**Keywords:** OCTA, Optical coherence tomography angiography, Epiretinal membrane, Visual acuity

## Abstract

**Background:**

To compare Optical Coherence Tomography Angiography (OCTA) biomarkers in epiretinal membrane (ERM) eyes and normal eyes, to investigate the association of each biomarker with best corrected visual acuity (BCVA), and to identify their optimal cut-off values for various best correct visual acuity in ERM eyes.

**Methods:**

A cross-sectional observational population study of the right eye of 6011 participants aged above 50 was performed. Central subfield thickness (CST_A), foveal avascular zone (FAZ_A), mean vessel density (mVD_full) and mean capillary perfusion density(mCPD_full) were measured by OCTA. Welch’s T-test was used to compare different mean values of parameters between normal eye and epiretinal membrane eyes. Multivariable linear regression test was used to assess the correlation between BCVA LogMAR and OCTA parameters. The cut-off values of biomarkers for best corrected visual acuity (BCVA LogMAR ≥ 0.18 / 0.3) in epiretinal membrane eyes were determined using receiver operating characteristic (ROC) and Area under curve (AUC) analysis.

**Results:**

ERM eyes had a significantly higher CST_A than normal eyes (Normal: 249.437 (95% CI 248.845 to 250.029) vs. ERM: 308.202 (95% CI 299.915 to 316.489) *P* < 0.001) A smaller FAZ_A was found in ERM eyes than normal eyes (Normal: 0.269 (95%CI 0.266 to 0.273) vs. ERM: 0.225 (95%CI 0.202 to 0.248) *P* < 0.001). Both mVD_full and mCPD_full were lower in ERM eyes when compared with normal eyes (mVD_full Normal: 16.397 (95% CI 16.343 to 16.451) vs. ERM 15.610 (95% CI 15.372 to 16.048) (*P* < 0.001)) (mCPD Normal: 0.399 (95% CI 0.397 to 0.400) ERM: 0.381 (95% CI 0.373 to 0.390) (*P* < 0.001)). Among CST_A, FAZ_A, mVD_full and mCPD_full, only CST_A was found to be significantly associated with best corrected visual acuity (BCVA). Better visual outcome is associated with smaller CST_A (beta* =0.265). CST_A was found to have an acceptable AUC value for BCVA LogMar ≥ 0.3 in epiretinal membrane eyes (AUC value = 0.708). The AUROC values of other biomarkers were less than 0.7 in predicting BCVA in epiretinal membrane eyes.

**Conclusion:**

CST_A was found to be thicker in ERM eyes while mVD_full, mCPD_full and FAZ_A were lower in ERM eyes than normal eyes. CST_A was found to be significantly associated with BCVA in ERM eyes. The cut off value of CST_A was found to be 345.5, with an acceptable AUC value for discriminating BCVA LogMAR ≥ 0.3 in epiretinal membrane eyes.

## Background

Epiretinal membrane (ERM) is a fibrocellular layer that is on the internal limiting membrane, associated with proliferation of myofibroblastic cells and extracellular matrix after posterior vitreous detachment [1, 2]. ERMs are composed of two layers including outer layer composed of non-cellular extracellular matrix proteins and inner layer of epiretinal cells. Causes of ERM include primary (idiopathic) and secondary causes such as trauma, iatrogenic, post retinal laser, diabetes mellitus, retinal vascular disease, chronic macula edema, uveitis, retinal detachment, intraocular tumours etc [2]. Management of epiretinal membrane mainly divided into conservative and surgical treatment [1]. Surgical treatment by three port pars plana vitrectomy with membrane peeling is the standard of care for ERM, however, the optimal timing of surgery is still unknown [1].

ERM’s symptoms can range from asymptomatic to severe visual loss with metamorphopsia. The severity of symptoms depend on various factors including their location, severity and duration [3]. Early stage ERM may not cause clinically significant reduction in visual acuity. Over time, it may increase its contractile properties and exert centripetal and anteroposterior traction on the retina [4]. This may result in damage to retinal neurons, reduction in perifoveal circulation, disruption of IS/OS and macular morphologic changes [5–8]. Various intraretinal and epiretinal biomarkers were demonstrated in the evaluation and histopathologic characterization of ERM in previous studies [6–13]. Total foveal thickness and visual acuity were demonstrated to have a significant correlation in previous studies [6–9]. Both inner and outer foveal retina are significantly thicken in ERM eyes, with inner foveal retina greater than outer foveal retina [6]. The outer retinal thickness has a greater effect in visual impairment in ERM eyes than inner retinal thickness [6]. Hyperreflective retinal spots on OCT in ERM eyes were shown to be associated with the degree of retinal thickening and visual acuity reduction (f). The presence of hyperreflective retinal spots may represent migration of retinal pigmented epithelial cells, lipid laden macrophages or photoreceptors degeneration in ERM eyes [11]. Presence of high proportion of gliotic tissue was found to have a significant effect on the surgical outcomes in Stage 2 ERM eyes as it may increase contractility and adherence of ERM to retina [12].

OCTA (Optical Coherence Tomography Angiography) is a novel non-invasive diagnostic method for detection of retinal and choroidal diseases [14]. It allows visualization of retinal vasculature without the need for injection of dye. Dense volumetric scans are used to provide details of blood flow in retina and choroidal vasculature. It uses multiple B-scans at the same location and compare the intensity and phase information to detect moving red blood cells. By performing volumetric scans and analysing the difference in signal between B-scans, angiogram can be created. It also has the ability to create quantitative analysis of the foveal avascular zone area (FAZ_A) and optic nerve head [15]. Furthermore, it can explore superficial and deep peripapillary plexus easily and separately using en face images. Apart from the aforementioned morphological changes in ERM eyes, ERM may induce traction on the retinal surface, which may lead to hemodynamic abnormalities in the retina [13]. Previous studies had demonstrated idiopathic epiretinal membrane may result in a smaller foveal avascular area (FAZ), higher superficial capillary plexus (SCP) and deep capillary plexus (DCP) and vessel density (VD) in foveal region, but lower SCP, DCP and VD in parafoveal area when compared with healthy eyes or postoperative eyes [13, 16–20]. However, most of them were limited by small sample size. Our study is an observational cross-sectional study with a large sample size which aims to observe the difference in OCTA parameters between normal and ERM eyes. Associations between different biomarkers and visual outcome were investigated.

## Methods

Our study collected data from the University of Hong Kong’s (HKU) Southern District Community Eye Screening Program, from 1/6/2021 to 17/5/2024. Participants living in Southern district of Hong Kong and aged 50 years old or above were included in the screening program. Participants were invited to perform a series of eye examinations for free on a one-off basis. Their result from eye examinations were interpreted by Ophthalmologists from the HKU Department of Ophthalmology. Ophthalmologists were invited to determine if participants have a macula problem based on participants’ clinical examination findings, fundus photo and spectral domain Optical Coherence Tomography (OCT) macula. Exclusion criteria for our study include participants with retinal or macula problem except epiretinal membrane. These include retinal drusen, retinal scar, subretinal fluid, retinal hemorrhage, epiretinal membrane, macular hole, atrophy, myopia maculopathy, diabetic mellitus retinopathy, peripheral degeneration, retina break, retinal vein occlusion and those with missing OCT angiography parameters. The right eye of each participant was selected for statistical analysis to avoid potential bias between both eyes. Our study was approved by the HKU/HA/Western Cluster Institutional Review Board (UW18516) and abides by the principles of the *Declaration of Helsinki*. All participants provided informed consent by signing a consent form.

Our OCTA examinations were done by trained clinical staff using the Carl Zeiss Cirrus 5000 machine (Carl Zeiss Meditec, Inc, Dublin, CA, USA) with Angioplex [21]. Our staff first aligned patient’s iris with the machine and advanced the scan head until the fundus came into view. During the process, they would check the optic nerve head was clearly visible. OCT alignment was ensured by centering the video image and scan region on the pupil and scan head towards the patient. In order to optimize the fundus video image, the working distance, polarization, Z offset and focus were adjusted before the capture of the scan. To reduce motion artifacts, the FastTrac retinal tracking technology was used to automatically track eye movement. All the scans were checked by our trained clinical staff any possible segmentation error. Manual adjustment of the segmentation lines after the scan would be done to correlate with the macula’s precise location. En face OCTA images were then generated as the software analyzes the scan using optical microangiography algorithm.

**Fig. 1:**
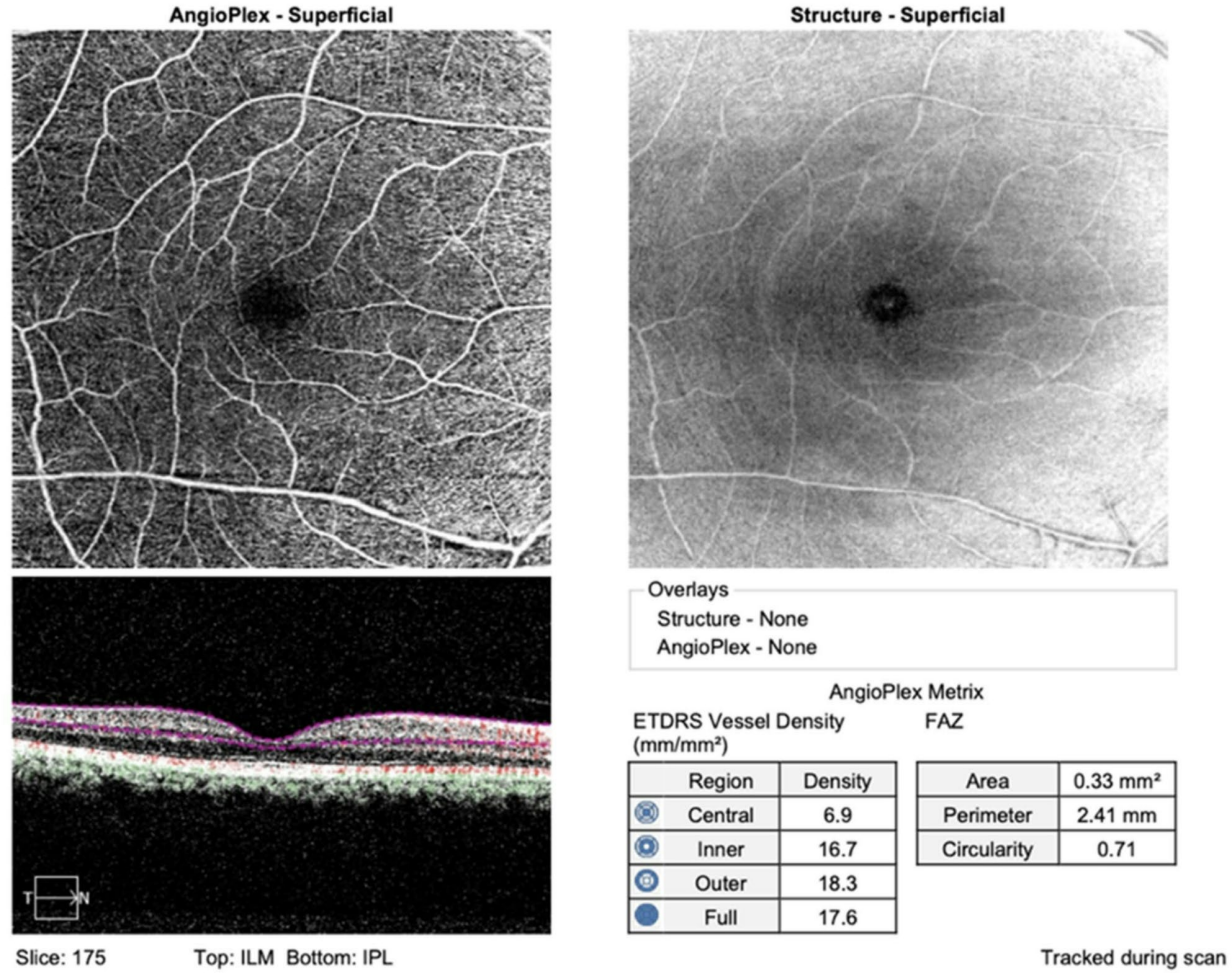
Images and Biomarkers obtained with Carl Zeiss Cirrus 500 machine (ILM-Internal limiting membrane, IPL-Inner plexiform layer, FAZ-Foveal acascular zone, ETDRS-Early Treatment diabetic retinopathy study)

To identify the foveal avascular zone area in the superficial capillary plexus, a real time eye tracking system with a 6 × 6 mm scanning model was used (Fig. 1). Definition of superficial capillary plexus is the distance between inner plexiform layer and the inner limiting membrane [22]. Our OCTA parameters were measured from the superficial capillary plexus. During the scan, multiple B scans at the same location was obtained by the OCT system. Moving blood cells were detected by comparing both phase information and intensity between the multiple B scans. Therefore, the OCTA algorithm can identify areas of flow and areas of static tissue, creating the angiogram. All the OCTA images were assessed by our trained clinical staff for quality control. Only images with signal strength index of greater than 7 were included for analysis [23]. Different ways can be used to visualize angiogram data, including two-dimensional view of the retinal or choroidal vasculature at a specific depth by En face OCTA images or three-dimensional view of vasculature by a stack of enface images.

Statistical analysis was done using IBM SPSS (version 31.0.2.0) and Microsoft excel. The mean is expressed as mean with 95% confidence Interval (CI). Our study’s endpoint include four OCTA biomarkers (FAZ_A, CST_A, mVD_full and mCPD_full). Welch’s T-test was used to compare different mean values of parameters between normal eye and epiretinal membrane eyes, p value was adjusted by Benjamini-Hochberg test. Multivariable linear regression test was used to assess the correlation between BCVA LogMAR and OCTA parameters. The cut-off values of biomarkers for best corrected visual acuity (BCVA LogMar ≥ 0.18 / 0.3) in epiretinal membrane eyes were determined using receiver operating characteristic (ROC) and Area under curve (AUC) analysis. A value of *p* < 0.05 is considered as statistically significant.

## Results

A total of 7542 participants and 7542 right eyes with OCTA examinations were recruited in this study. After screening of the recruited participants, 1531 eyes were excluded due to macula pathology or incomplete data. The mean age of all of the included participants was 62.575 (95% CI: 62.377, 62.773),. Male to female ratio was 0.550. Best corrected visual acuity (BCVA) LogMAR was 0.05 (95% CI: 0.049, 0.055) with the spherical equivalent − 0.64 (95% CI: -0.699, -0.572) dioptre. Baseline demographic, ocular characteristics of all participants were displayed in (Table 1). Among the included 6011 participants, 5769 of them were categorised into normal macula in their right eye while 242 of them were found to have epiretinal membrane in their right eye (Table 2).

**Table 1:** Demographic and ocular characteristics of all participants

Parameter	Number	Mean	95% Confidence Interval
Age	6011	62.575	62.377, 62.773
Male vs. Female	2132 vs. 3879	NA	NA
BCVA (LogMar)	6011	0.05	0.049, 0.055
Spherical Equivalent (dioptre)	6011	-0.636	-0.699, -0.572
CST_A	6011	251.8	251.083, 252.523
FAZ_A	6011	0.2676	0.264, 0.271
mVD_Full	6011	16.369	16.312, 16.423
mCPD_full	6011	0.398	0.396, 0.399

**Table 2:** Mean value of demographic and ocular characteristics of normal right eye and epiretinal membrane right eye

Parameter	Normal			ERM			Adjusted *P* value
	Number	Mean	95% Confidence Interval	Number	Mean	95% Confidence Interval	
Age	5769	62.410	62.208, 62.612	242	66.504	66.570, 67.437	< 0.001
Male vs. Female	5769	M = 2043		242	M = 89		0.664
BCVA (LogMar)	5769	0.0497	0.0468, 0.0526	242	0.1067	0.090, 0.123	< 0.001
Spherical Equivalent	5769	-0.620	-0.684, -0.555	242	-1.002	-1.372, -0.623	0.030
CST_A	5769	249.437	248.845, 250.029	242	308.203	299.915, 316.489	< 0.001
FAZ_A	5769	0.269	0.266, 0.273	242	0.225	0.202, 0.248	< 0.001
mVD_Full	5769	16.397	16.343, 16.451	242	15.610	15.372,16.048	< 0.001
mCPD_full	5769	0.399	0.397, 0.400	242	0.381	0.373, 0.390	< 0.001

By comparing the mean value of normal eyes and ERM eyes, a significant older age was found in RE ERM participants when compared with RE normal macula participants (62.410 vs. 66.504, *P* < 0.001). A significant more minus in spherical equivalent was also demonstrated in RE ERM participants than RE normal macula participants (*P* = 0.0303). CST_A was found to be higher in ERM eyes than normal macula eyes (308.203 vs. 249.437, *P* = 0.000). For FAZ_A, ERM eyes were significantly smaller than normal eyes. Both mVD_full and mCPD_full were found to be significantly lower in ERM eyes than normal eyes (Table 2).

Among all the RE ERM participants, multivariable linear regression analysis was performed to investigate any associations between OCTA parameters and BCVA LogMAR (visual outcome) (Table 3). From our results, no significant correlation was demonstrated between FAZ_A, mVD and mCPD BCVA LogMAR in ERM eyes. Significant correlations were demonstrated when CST_A. CST_A had a significant positive beta coefficient with BCVA LogMaR (*P* < 0.01).

**Table 3:** Multivariable regression analysis of OCTA biomarkers to explore correlation with BCVA in ERM eyes

Independent Parameters	Dependent Parameters (BCVA LogMAR)		
	Standardized Coefficients Beta*	Unstandardized B (95% CI interval)	*P* Value
Age	0.372	0.007 (0.004, 0.009)	< 0.001
Gender (Male vs. Female)	0.126	0.034 (0.004, 0.065)	0.028
Spherical Equivalent	-0.141	-0.007 (-0.013, -0.001)	0.033
CST_A	0.265	0.001 (0.000, 0.001)	< 0.001
FAZ_A	0.028	0.020 (-0.064, 0.104)	0.639
mVD_full	-0.859	-0.042 (-0.112, 0.028)	0.239
mCPD_full	0.749	1.447 (-1.310, 4.205)	0.302

### ROC analyses of significantly associated OCTA parameters for predicting BCVA LogMAR in epiretinal membrane eyes

Despite there were no worldwide accepted guidelines at this moment, surgical treatment for ERM eyes were traditionally considered when visual acuity was 20/40 or 20/30 [24–26]. We determine the optimal cut-off points of OCTA parameters for estimating BCVA by the maximum Youden’s index.

### ROC analyses of OCTA parameters for estimating BCVA LogMAR ≥ 0.18

**Fig. 2:**
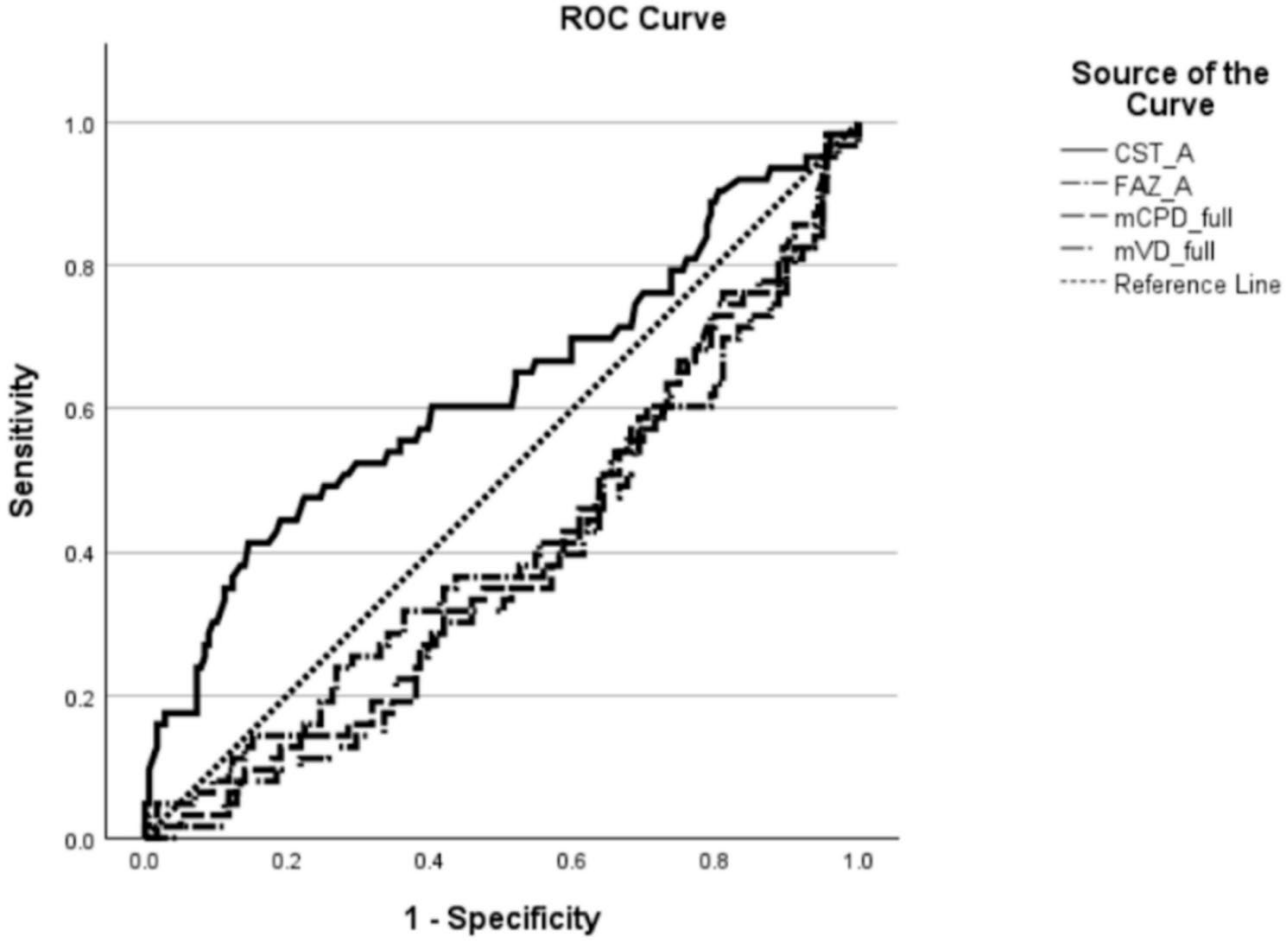
ROC analysis of FAZ_A, mVD_full, mCPD_full, CST_A for BCVA LogMar ≥ 0.18

Four OCTA parameters, FAZ_A, mVD_full, mCPD_full and CST_A were analyzed in our study. (Table 4; Fig. 2). The AUC of all four indices were found to be significant (*P* < 0.05). For CST_A, the AUC was found to be 0.625 (Fig. 2) with the cut-off point found to be 351. The AUC of FAZ_A was 0.413 while the AUC of mVD_full was 0.384. The AUC of mCPD_full was found to be 0.394. All of four parameters’ AUC value did not reach 0.7.

**Table 4:** The optimal cut points of OCTA parameters for BCVA LogMar ≥ to 0.18

OCTA parameters	Cut-off point	Sensitivity	1-Specificity	Youden’s Index	Area	*P* value	Std. Error of area	95% Confidence interval
FAZ_A	0.5304	0.048	0.017	0.031	0.413	0.044	0.043	0.328, 0.498
mVD_full	10.1798	0.984	0.961	0.023	0.384	0.004	0.040	0.305, 0.462
mCPD_full	0.2424	0.984	0.955	0.029	0.394	0.009	0.041	0.314, 0.474
CST_A	351	0.413	0.145	0.267	0.625	0.005	0.044	0.538, 0.712

### ROC analyses of OCTA parameters for estimating BCVA LogMAR ≥ 0.3

**Fig. 3:**
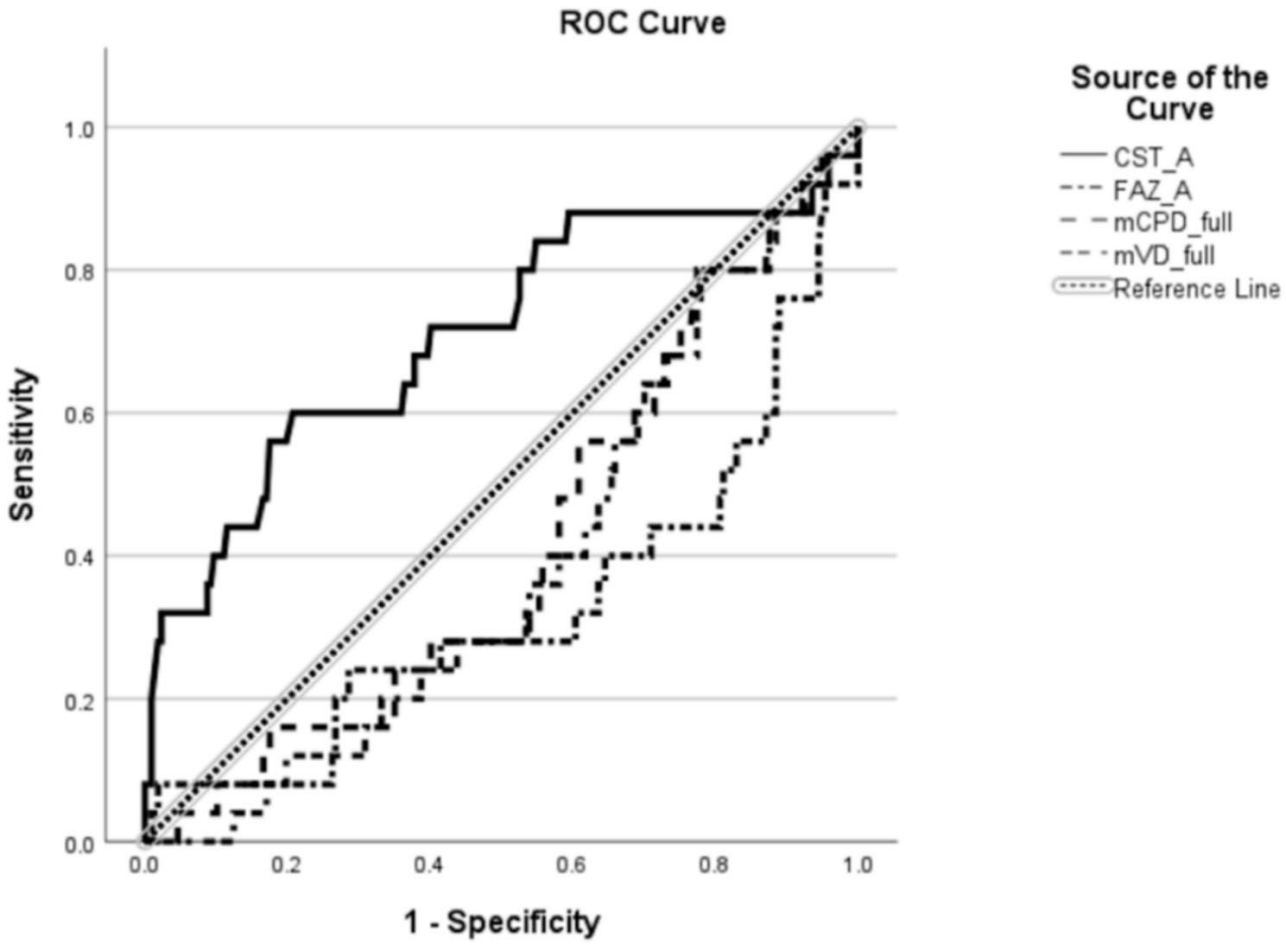
ROC analysis of FAZ_A, mVD_full, mCPD_full, CST_A for BCVA LogMAR ≥ 0.3

The AUC of all four indices were found to be significant (*P* < 0.05). (Table 5; Fig. 3) For CST_A, the AUC was found to be 0.708 (Fig. 2) with the cut off point found to be 345.5. The AUC of FAZ_A was 0.329 while the AUC of mVD_full was 0.390. The AUC of mCPD_full was found to be 0.413. The cut off value of CST_A was found to have an acceptable AUC value for estimating BCVA LogMAR ≥ 0.3 in ERM eyes.

**Table 5:** The optimal cut points of OCTA parameters for BCVA LogMAR ≥ 0.3

OCTA parameters	Cut-off point	Sensitivity	1-Specificity	Youden’s Index	Area (AUC)	*P* value	Std. Error of area	95% Confidence interval for Area
FAZ_A	0.5304	0.080	0.018	0.062	0.329	0.08	0.064	0.204, 0.454
mVD_full	14.2491	0.800	0.774	0.026	0.390	0.039	0.053	0.285, 0.494
mCPD_full	0.3422	0.800	0.779	0.021	0.413	0.128	0.057	0.301, 0.525
CST_A	345.5	0.600	0.207	0.393	0.708	0.001	0.064	0.582, 0.834

## Discussion

Our study is the first observational cross-sectional study with a vast sample size investigating OCTA biomarkers in epiretinal membrane eyes in Hong Kong. Our study also investigated in the associations between OCTA biomarkers and best corrected visual acuity in ERM eyes.

We have demonstrated that ERM eyes had a significantly smaller FAZ_A than normal eyes, this finding is similar to previous studies [27, 28]. The proposed mechanism of smaller FAZ in ERM eyes than normal eyes is due to the afferent contraction of ERM on the outer layer of retina nearby the foveal center. The contraction force by ERM may result in changes both vertically and horizontally [29]. ERM development leads to anteroposterior forces leading to macular thickening and tangential forces leading to vessel displacement. This is further demonstrated by by Yoshida et al. who found an inverse correlation between FAZ_A and the stage of ERM. The stage of ERM used in the aforementioned study was suggested by Govetto et al., which ERM was graded by ss-OCT macula scans by the morphology of the foveal pit and retinal layers [29]. According to the study, vertical morphological changes include disruption of retinal layers and change in ectopic inner retinal layers’ appearance. As ERM progresses, there will be contraction of retinal surface, resulting in contraction of FAZ or retinal folds horizontally. This may also explain CST_A is thicker in ERM eyes than normal eyes, as demonstrated in our study. To evaluate the effect of ERM / internal limiting membrane on the foveal structure and vasculature in eyes, previous studies had performed OCTA in ERM eyes before and after vitrectomy with ILM membrane peeling [14, 20, 29, 30]. The peculiar behavior of the FAZ area was well known after ILM peeling. Bacherini et al. had demonstrated a reduction of FAZ after peeling at 1 month follow up and a gradual enlargement afterwards [14]. This is due to the creation of centrifugal intrinsic force by ILM on the retina. Centripetal movement will be generated by the peeling of ILM, hence relieving the centrifugal intrinsic force. Another study by Caretti et al. measured FAZ perimeter before and after ERM surgery at 1 and 6 month intervals. FAZ perimeter pre-operation was found to be significantly smaller than 6 months after surgery [31]. We should aware that FAZ_A measurement may have a chance of biased in OCTA. The reason is that ERM eyes may have more retinal folding and disorganizations, resulting in false retinal layer segmentation, superficial capillary plexus crowding and elevation [31]. Furthermore, the measurement may be affected by misalignment of OCTA and varied platform-specific FAZ algorithms.

The most common symptoms in ERM eyes were metamorphopsia and blurring of vision. The above symptoms were two major considering factors to decide whether ERM surgery is indicated. Multiple previous studies have tried to investigate into whether FAZ_A can predict symptoms of metamorphopsia, but the results varied [19, 27, 29]. Both Kitagawa et al. and Yoshida et al. compared preoperative and postoperative ERM eyes’ OCTA parameters and found no significant association between FAZ_A and metamorphopsia [19, 27]. However, other studies demonstrated a significant association between metamorphopsia and FAZ_A [29, 32]. Another important decision-making factor in deciding when to operate on ERM is the visual acuity of the patient. From our study, we have demonstrated an insignificant association between FAZ_A and visual acuity in ERM patients. Similar findings were demonstrated by Shiihara et al., which also demonstrated a non-significant relationship between BCVA and FAZ parameters [32]. Bacherini et al. investigated in 23 ERM eyes, with OCTA done preoperatively and postoperatively [14]. Significant association between FAZ_A and visual acuity was found at baseline and 1 month post operation, but it is limited by its small sample size. The underlying mechanism of the varied results of FAZ_A may be due to the underlying pathology and causes of ERM, Romano et al. compared ERM caused by idiopathic or diabetes mellitus, and found the perifoveal capillary free zone measured by OCTA were significantly increase after ILM peeling in diabetic caused ERM, but no significant change in idiopathic ERM eyes [33]. Regarding the varied conclusions in association of FAZ_A with metamorphopsia and visual acuity, further studies may be needed to investigate whether FAZ_A is a suitable biomarker to act as a predictive factor for visual function in ERM eyes or as an indicator for ERM surgeries.

In our study, we discovered that by comparing ERM and normal eyes, both mVD_full and mCPD_full were found to be significantly lower in ERM eyes than normal eyes. Hsia et al. had investigated into the macula retinal vasculature in different severity of idiopathic epiretinal membrane eyes. The results showed that more severe ERM eyes had an increase in average vessel diameter, decrease in skeleton density and vessel tortuosity [34]. Retinal vasculature includes superficial and deep capillary plexus at the retinal nerve fibre layer and retinal ganglion cell layer. The two capillary plexus supply nutrients to the inner retina. The tangential tractional force of ERM may result in changes at the inner retinal structures and inner retinal vasculature. Apart from the tractional force, ERM may also demonstrate a compressive stress on the retina, resulting in the diminished microvasculature and stagnated blood flow [34, 35]. Kim et al. investigated into the temporal microvasculature changes of parafoveal area in ERM eyes before and after ERM surgeries. He suggested that blood flow of deep capillary plexus are more affected by presence of ERM than superficial capillary plexus as the deep capillary plexus is supplied by superficial capillary plexus’s vertical branches [35]. Caretti et al. suggested no significant change of foveal vessel density and parafoveal vessel density at superficial capillary plexus in preoperative and postoperative ERM eyes, but a lower mean vessel density in postoperative eyes. The study emphasized the elevation and crowding of vessels in superficial capillary plexus are varied in different patient samples with regard to the severity of the ERM pathology. This might result in a falsely augmented density in OCTA parameters [31]. Another minor attributing factor may be more retinal folding and disorganization in ERM eyes, resulting in difficulty to detect thoroughly retinal capillary network by OCTA [34]. The foveal architecture distortion may lead to false retinal layer segmentation and thus controversial vessel quantification measurements. Previous studies have found segmentation errors up to 69% in ERM eyes [36, 37]. In particular, the inner retinal layers such as the inner plexiform layer are most prone to inaccurate segmentation, which are also the most important for quantification of the capillary plexus.

CST_A was found to be significantly to be higher in ERM eyes than normal eyes while mVD_full and mCPD_full were significantly lower in ERM eyes. Significant associations between CST_A and BCVA LogMAR was demonstrated. However, mVD_full and mCPD_full was found to be insignificantly associated with BCVA. Bacherini et al. demonstrated a significant increase in the superficial capillary plexus vessel density and perfusion density in postoperative ERM eyes. In the baseline preoperative ERM eyes, lower perfusion or vessel density of retinal and choroidal plexi were associated with poor visual acuity [14]. Study by Feng had investigated 30 ERM eyes before and after operation, showing vessel density of foveal deep capillary plexus was a positive prognostic factor for post operative visual outcome and macula sensitivity [38]. Xu et al. also demonstrated parafoveal vessel density was a predictor of visual function after ERM surgery [39]. The different between our study from the abovementioned study is that our study had a greater sample size and we solely investigated in ERM eyes only, instead of comparing pre and post ERM operation. We should be aware that our study’s biomarkers were generated from superficial capillary plexus. Further studies may be needed to further investigate in the associations between visual acuity and OCTA biomarkers from deep capillary plexus.

One of the major aims of our study is to find the cut off values of each OCTA biomarkers. CST_A was found to have an acceptable AUC value for estimating BCVA LogMAR ≥ 0.3 in ERM eyes. However, other parameters were found to be suboptimal to create a cut off values for BCVA LogMAR ≥ 0.3 / 1.8. This shows that CST_A may be used as an additional indicator to provide surgeons on the decision on performing ERM surgery on a patient. This also provide insight into the application of OCTA for ERM patients in daily clinical practice. Our study only included associations between the biomarkers and visual acuity but did not investigate into associations between biomarkers and patients’ metamorphopsia symptoms. Further studies are needed to conclude on the role of OCT_A biomarkers in ERM eyes on metamorphopsia in ERM eyes.

Indications for pars plana vitrectomy and membrane peeling surgery of ERM eyes were largely dependent on ophthalmologists’ subjective assessment and there were no worldwide accepted guidelines at this moment [31]. The advancement in OCTA provides a potential role to provide an objective data for surgical decision making. Apart from only using perfusion data, recent studies had given insights in the complementary value of OCTA-derived parameters with biochemical cytokine profiling in vitreoretinal disorder, which might be an interesting area for further exploration [40, 41]. Ruggeri et al. analyzed the microRNA profiling in vitreous humor/ serum and OCTA imaging in vitreomacular interface disease or rhegmatogeneous retinal detachment. The results showed different expression of miRNA had a significant correlation with superficial capillary plexus macular vessel density [40]. Another study suggested several miRNAs had found to be significantly higher in vitreous of proliferative diabetic retinopathy eyes than macula hole eyes. Multiple studies had investigated in the change in macular microcirculation change by OCTA in rhegmatogenous retinal detachment repaired eyes, demonstrating an enlarge of FAZ in deep capillary plexuses and reduction of vascular density [42]. Further study had investigated in preretinal abnormal tissue and its association with vascular changes by OCTA in macula- on rhegmatogenous retinal detachment postoperative eyes, suggesting endolaser area had a significant effect on presence of preretinal abnormal tissue, which might influence future ERM development [10]. The developing evidence of studies delineated the role of macula micro-perfusion and its relationship with tractional remodeling / functional outcome. This may further refine the prognostic assessment of structural, vascular imaging biomarkers and inflammatory mediators in ERM or related vitreoretinal conditions in the future.

The major advantage in our study is that our study has a large sample size. A total of 6011 eyes were included in our study, with 242 of them were classified to have epiretinal membrane. This may have a better power in determining the associations between biomarkers and their best visual acuity in ERM eyes. The major limitation is that our study is a cross- sectional study, we were not able to investigate into any significant changes of the above biomarkers between pre and post ERM operated eyes. Our study only includes non-operated ERM eyes. Another limitation is that despite our study excluded eyes with other retinal or macula problem, we did not count lens status and systemic vascular/diabetes status of our patients in our study, which is a common factor in affect a patients’ visual acuity. Also staging of ERM is not included in our analysis. However, with the large sample size in our study, we believe the effect of the above condition can be lowered.

## Conclusion

CST_A was found to be thicker in ERM eyes while mVD_full, mCPD_full and FAZ_A were lower in ERM eyes than normal eyes. CST_A was found to be significantly associated with BCVA in ERM eyes. The cut off value of CST_A was found to be 345.5 with an acceptable AUROC value for BCVA LogMAR ≥ 0.3 in epiretinal membrane eyes.

## Data Availability

Data available by request to corresponding author up to 3 years after publication.

## Abbreviations

CST_A: Angiography- Central Subfield Thickness (µm)
FAZ_A: Foveal avascular zone Area (mm2)
mVD_Full: Mean Vessel Density (mm/mm2) Full
mVD_C: Mean Vessel density center(mm/mm2)
mCPD_full: Mean capillary perfusion density full
